# Hypoglycemia-induced changes in complement pathways in type 2 diabetes

**DOI:** 10.1016/j.athplu.2021.11.002

**Published:** 2021-11-18

**Authors:** Abu Saleh Md Moin, Manjula Nandakumar, Ilhame Diboun, Ahmed Al-Qaissi, Thozhukat Sathyapalan, Stephen L. Atkin, Alexandra E. Butler

**Affiliations:** a Diabetes Research Center (DRC), Qatar Biomedical Research Institute (QBRI), Hamad Bin Khalifa University (HBKU), Qatar Foundation (QF), PO Box 34110, Doha, Qatar; b Hamad Bin Khalifa University (HBKU), Doha, Qatar; c Academic Endocrinology, Diabetes and Metabolism, Hull York Medical School, Hull, UK; d Leeds Medical School, Leeds, UK; e Royal College of Surgeons in Ireland Bahrain, Adliya, Bahrain

**Keywords:** Hypoglycemia, Type 2 diabetes, Complement proteins, Proteomics

## Abstract

**Background and aims:**

An association between hypoglycaemia and adverse cardiovascular events has been suggested from longitudinal and retrospective cohort studies. The complement pathway proteins in hypoglycemia are not well studied. Here, we hypothesized that these circulating proteins would be elevated in response to hypoglycemia in type 2 diabetes (T2D) through the inflammatory response.

**Methods:**

A prospective, parallel study in T2D (n = 23) and controls (n = 23). Subjects underwent insulin-induced hypoglycemia with blood sampling at baseline, hypoglycemia and post-hypoglycemia; SOMAscan proteomic analysis of complement pathway-related proteins, cytokines and inflammatory proteins was undertaken.

**Results:**

At baseline: Complement C2 (p < 0.05) and Factor B (p < 0.05) were elevated in T2D. At hypoglycemia: Complement C2 (p < 0.05) and Factor B (p < 0.01) remained elevated, whilst Factor I became elevated (p < 0.05) in T2D; Complement C4b became elevated in controls (p < 0.05). In the post-hypoglycemia follow up period, Complement C2, Factor B and Factor I remained elevated in T2D; in addition, Factor D, Factor H and mannose-binding protein C showed elevations in T2D, whilst properdin, complement C3b, Factor H-related protein 5, complement C1q and decay-accelerating factor (DAF) showed elevations in controls. Granger causality analysis showed that inflammatory proteins appeared to drive complement protein changes in T2D; conversely, in controls, complement proteins drove inflammatory protein changes.

**Conclusions:**

Baseline elevations in C2 and Factor B indicate upregulation of the complement pathway in T2D. Changes in complement pathway-related protein levels in response to hypoglycemia suggest both intrinsic and alternative pathway activation at 2-h that appears driven by the underlying inflammation in T2D and could contribute to a cardiovascular event.

ClinicalTrials.gov NCT03102801. Date of registration April 6, 2017, retrospectively registered. https://clinicaltrials.gov/ct2/show/NCT03102801?term=NCT03102801&draw=2&rank=1.

## Highlights

• Complement pathway protein changes in hypoglycemic subjects not well studied.
• Complement response to hypoglycemia in type 2 diabetes (T2D) and controls studied.
• Baseline: C2 and Factor B elevated in T2D; at hypoglycemia: Factor I also elevated.
• Post-hypoglycemia in T2D, Factors D and H plus protein C additionally elevated.
• In T2D inflammatory proteins drive complement proteins; opposite found in controls.

## Introduction

Results emanating from a number of large randomized controlled studies designed to investigate the effect of improving glycemic control on vascular complications in type 2 diabetes (T2D) are in accord with finding an association between severe hypoglycaemia and adverse events, notably cardiovascular-related events, and increased mortality [[1], [2], [3], [4], [5]]. The action to control cardiovascular risk in diabetes (ACCORD) study was terminated prior to completion due to an increase in mortality in the intensive glucose control arm in T2D patients [6], suggesting that the increased frequency of hypoglycemic events likely underlay the poor outcomes. Consistent with these findings of association between hypoglycaemia and adverse cardiovascular events were reports from a number of longitudinal and retrospective cohort studies [[7], [8], [9]] as well as a post hoc analysis of the Veteran Affairs Diabetes Trial (VADT) [10]; conversely, other studies, such as the Bypass Angioplasty Revascularization Investigation in Type 2 Diabetes study, failed to find such association [11]. Nevertheless, the weight of evidence has fostered significant concerns regarding the risk of hypoglycaemia and adverse cardiovascular outcomes in patients with T2D.

Patients with T2D have an increased risk of thrombotic disease and a number of mechanisms working in concert underlie this risk: coagulation factor activation [12], reduced fibrinolysis [13] and activated dysfunctional platelets [14]. Hypoglycemia, an adverse outcome of glucose-lowering medication in T2D that is escalated by tight glucose control regimens, provokes these mechanisms, there being an established relationship of hypoglycaemia with thrombogenicity [15] and platelet hyper-reactivity [16,17]. In addition to inhibiting fibrinolysis and activating platelets, hypoglycemia also induces pro-inflammatory proteins, such as the interleukins [[18], [19], [20]] and it is likely that recurrent hypoglycemic events further provoke thrombotic processes, such as atherosclerosis, in a cumulative manner through induction of the inflammatory response [21].

The inflammatory response is rapidly induced in response to hypoglycaemia and likely drives other acute response pathways, such as the complement cascade, with significant crosstalk between the two [22,23]. Complement system activation has been reported in obesity, insulin resistance and T2D [24] and has additionally been implicated in diabetic micro- and macrovascular complications [25,26]. Activation of the complement system has been reported in T2D patients diagnosed with myocardial infarction (MI), hyperactivation being associated with a poor prognosis [27].

While recognised as involved in the response to the hyperglycemia of diabetes, complement cascade pathways have not been well studied in the context of hypoglycaemia in T2D. Here, we hypothesized that hypoglycaemia would trigger an inflammatory response that would, in turn, activate complement pathways. Therefore, in this study, we determined a panel of circulatory complement pathway-related proteins over a 24-h timecourse in T2D and control subjects.

## Materials and methods

### Study design

As has previously been described [28], “This prospective parallel study was performed in 46 subjects, adult T2D (n = 23) and control (n = 23) at the Diabetes Centre at Hull Royal Infirmary; all subjects were Caucasian and between the ages of 40–70 years. In the T2D cohort, duration of diabetes was <10 years and all subjects were on a stable dose of medication (metformin, statin and/or angiotensin converting enzyme inhibitor/angiotensin receptor blocker) for the preceding 3 months. For inclusion in the T2D cohort, no glucose-lowering agents other than metformin were allowed, HbA1c levels were required to be <10% (86 mmol/mol)], and patients must not have had either hypoglycemic unawareness or hypoglycemia within the preceding 3-month period. In the control group, an oral glucose tolerance test was used to exclude diabetes. For study inclusion, subjects in both T2D and control cohorts were required to have a body mass index (BMI) between 18 and 49 kg/m^2^, normal renal and hepatic biochemical indices and no prior history of cancer nor any contraindication to insulin infusion to achieve hypoglycemia (ischemic heart disease, epilepsy, seizure history, drop attacks, history of adrenal insufficiency and treated hypothyroidism).

The trial was approved by the North West-Greater Manchester East Research Ethics Committee (REC number:16/NW/0518), registered at www.clinicaltrials.gov (NCT03102801) and conducted according to the Declaration of Helsinki.”

### Insulin infusion

The insulin infusion was performed as previously detailed [29]. “Following an overnight fast, bilateral ante-cubital fossa indwelling cannulas were inserted 30–60 min prior to the commencement of the test (0830h). To induce hypoglycemia, soluble intravenous insulin (Humulin S, Lilly, UK) was given in a pump starting at a dose of 2.5mU/kg body weight/min with an increment of 2.5mU/kg body weight/min every 15min by hypoglycemic clamp [30], until two readings of capillary blood glucose measured by a glucose analyser (HemoCue® glucose 201+) of ≤2.2 mmol/L (<40 mg/dl) or a single reading of ≤2.0 mmol/L (36 mg/dl) [30], after which the hypoglycemia was immediately reversed with an infusion of 10% dextrose. The blood sample schedule was timed subsequently in respect to the time point that hypoglycemia occurred. Following the identification of hypoglycemia, intravenous glucose was given in the form of 150 ml of 10% dextrose and a repeat blood glucose check was performed after 5 min if blood glucose was still <4.0 mmol/L”

### SOMA-scan assay

“The SOMAscan assay was used to quantify the complement pathway-related proteins, cytokines and inflammatory proteins reported in this study. The protein assay was performed on an in-house Tecan Freedom EVO liquid handling system (Tecan Group, Maennedorf, Switzerland) utilizing buffers and SOMAmers from the SOMAscan HTS Assay 1.3K plasma kit (SomaLogic, Boulder, CO) according to manufacturer’s instructions and as described previously [31,32]. The assay was performed in 96-well plates containing up to 85 plasma samples, 3 quality control and 5 calibrator plasma samples. Briefly, EDTA plasma samples were diluted into bins of 40%, 1% and 0.05% and incubated with streptavidin-coated beads immobilized with dilution-specific SOMAmers via a photocleavable linker and biotin. After washing bound proteins were first biotinylated and then released from beads by photocleaving the SOMAmer-bead linker. The released SOMAmer-protein complex was treated with a polyanionic competitor to disrupt unspecific interactions and recaptured on the second set of streptavidin-coated beads. Thorough washing was performed before 5’ Cy3 fluorophore labelled SOMAmers were released under denaturing conditions, hybridized on microarray chips with SOMAmer-complementary sequences, and scanned using the SureScan G2565 Microarray Scanner (Agilent, Santa Clara, CA).

#### Data processing and analysis

Initial Relative Fluorescent Units (RFUs) were obtained from microarray intensity images using the Agilent Feature Extraction Software (Agilent, Santa Clara, CA). Raw RFUs were normalized and calibrated using the software pipeline provided by SomaLogic. Samples with a high degree of hemolysis (Haptoglobin log RFU < 10) were excluded from the analysis.”

### Statistical analysis

There are no studies detailing the changes in complement pathway-related proteins in response to hypoglycaemia on which to base a power calculation. Sample size for pilot studies has been reviewed by Birkett and Day [33] They concluded that a minimum of 20 degrees-of-freedom was required to estimate effect size and variability. Hence, we needed to analyse the samples from a minimum of 20 patients per group. Comparison between groups was performed at each timepoint using Student’s t-test. A p-value of <0.05 was considered statistically significant. Within-group comparisons are as follows: changes from baseline, and from hypoglycemia, to each subsequent time point were compared using a mixed linear model performed using the R package ‘lmerTest’ (R version 4.0.2). Statistical analysis was performed using Graphpad Prism (San Diego, CA, USA). The resulting p-values were further subjected to multiple testing correction using the FDR procedure.

#### Partial correlation and Granger causality analysis

Partial correlations between inflammatory and complement proteins were calculated using the Graphical Marcov Model implemented in the GGM package in R. To assess causal relationships between inflammatory and complement proteins, we used the Granger test implemented in R package ‘lmtest’: Briefly, the test evaluates whether previous values of an inflammatory protein are predictive of future values of a complement system protein. For each pair of inflammatory and complement proteins, the test was also performed in the opposite direction. Pairs where the test was significant in both ways were ignored since causation could not possibly be inferred.

### Protein-protein interaction tools

STRING 11.0 (Search Tool for the Retrieval of Interacting Genes) was used to visualize the known and predicted Protein-Protein Interactions for proteins identified by SOMAscan assay in plasma of T2D versus control subjects (https://string-db.org/). Interactions between proteins are evidence-based and collated from databases, experiments, neighbourhood, gene fusion, co-occurrence, text mining, co-expression, and homology. Here, we determined the relationships between the complement pathway-related proteins presented in this study.”

## Results

### Demographic and biochemical characteristics of study participants

**Fig. 1:**
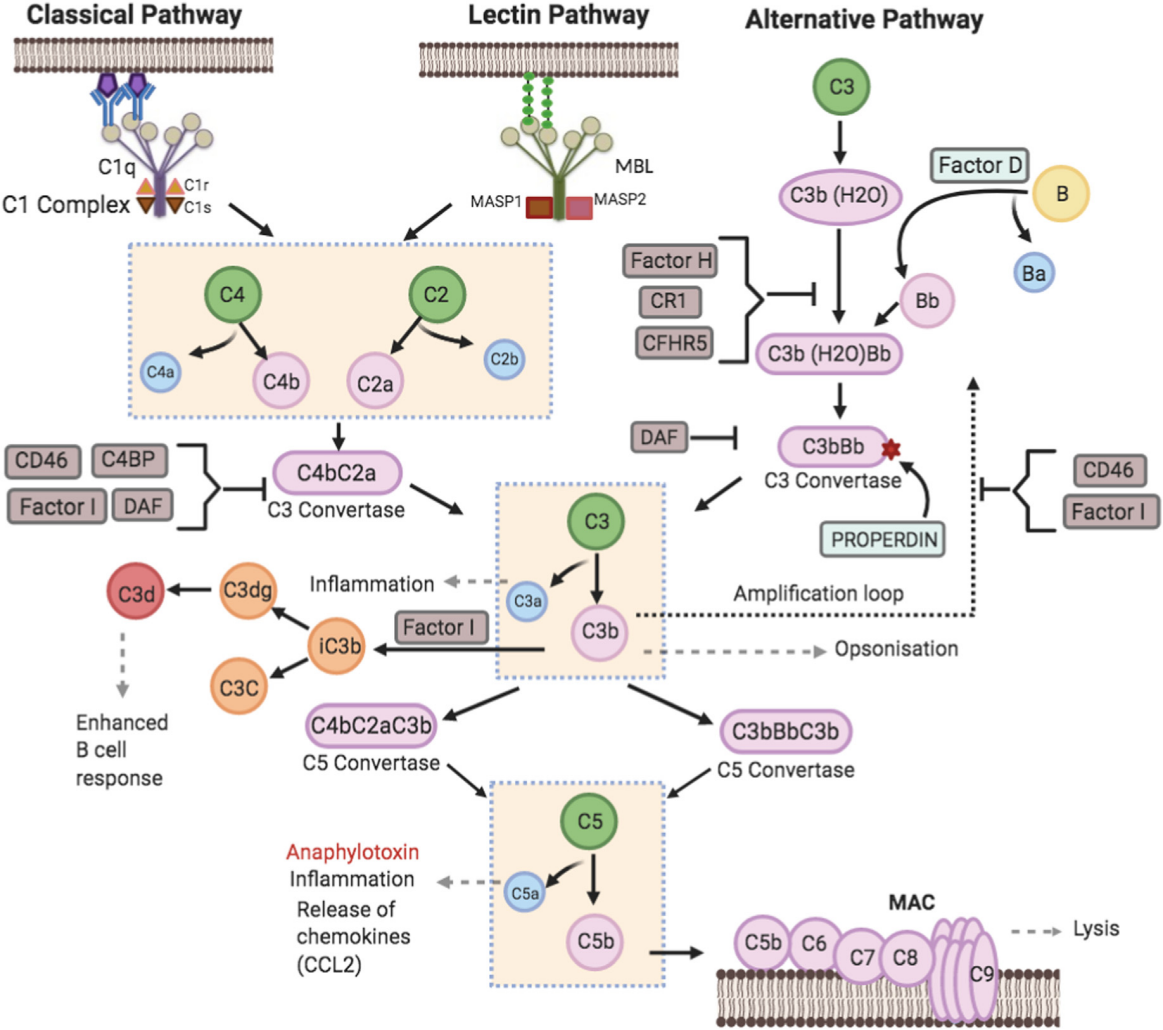
A schematic representation of the classical, lectin and alternative pathways of the complement system. In the classical pathway, C1q forms a complex with C1r and C1s leading to C3b production that opsonizes the pathogen. In the lectin pathway, the mannan-binding lectins (MBLs) lead to the activation of mannan-binding lectin serine proteases (MASPs), producing C4a and C4b and C2a and C2b, with subsequent steps in common with the classical pathway. The alternative pathway is initiated by spontaneous hydrolysis of the C3 molecule, leading to the amplification of C3b production and opsonisation of the pathogen. Subsequent production of C4bC2aC3b and C3bBbC3b results in C5b formation that binds to target cells and initiates recruitment of C6-9, creating a membrane attack complex (MAC) leading to target cell lysis. C4a, C3a, C5a are anaphylatoxins that when bound to immune cells receptors initiate local inflammatory responses and trigger cytokines and chemokines release.

46 subjects (23 individuals with T2D, 23 controls) were recruited for the study [29]. Relative to controls, the T2D cohort had an elevated BMI (32 ± 4 vs 28 ± 3 kg/m^2^, T2D vs control, p < 0.001). Twenty-four complement pathway-related proteins were included in the analysis and their interactions are depicted schematically in Fig. 1: Properdin, Complement C2, C3, C3a, C3b, inactivated C3b (iC3b), C3d, C4a, C4b, C5, C5a, C5b-6 complex, C8a, Factor B, Factor D, Factor H, Factor H-related 5, Factor I, C1q, C1r, mannan-binding lectin (MBL), mannan-binding lectin serine protease 1 (MASP1), Complement decay-accelerating factor (CD55 or DAF) and C–C motif chemokine ligand 2 (CCL2).

### Differences between T2D and controls

**Fig. 2:**
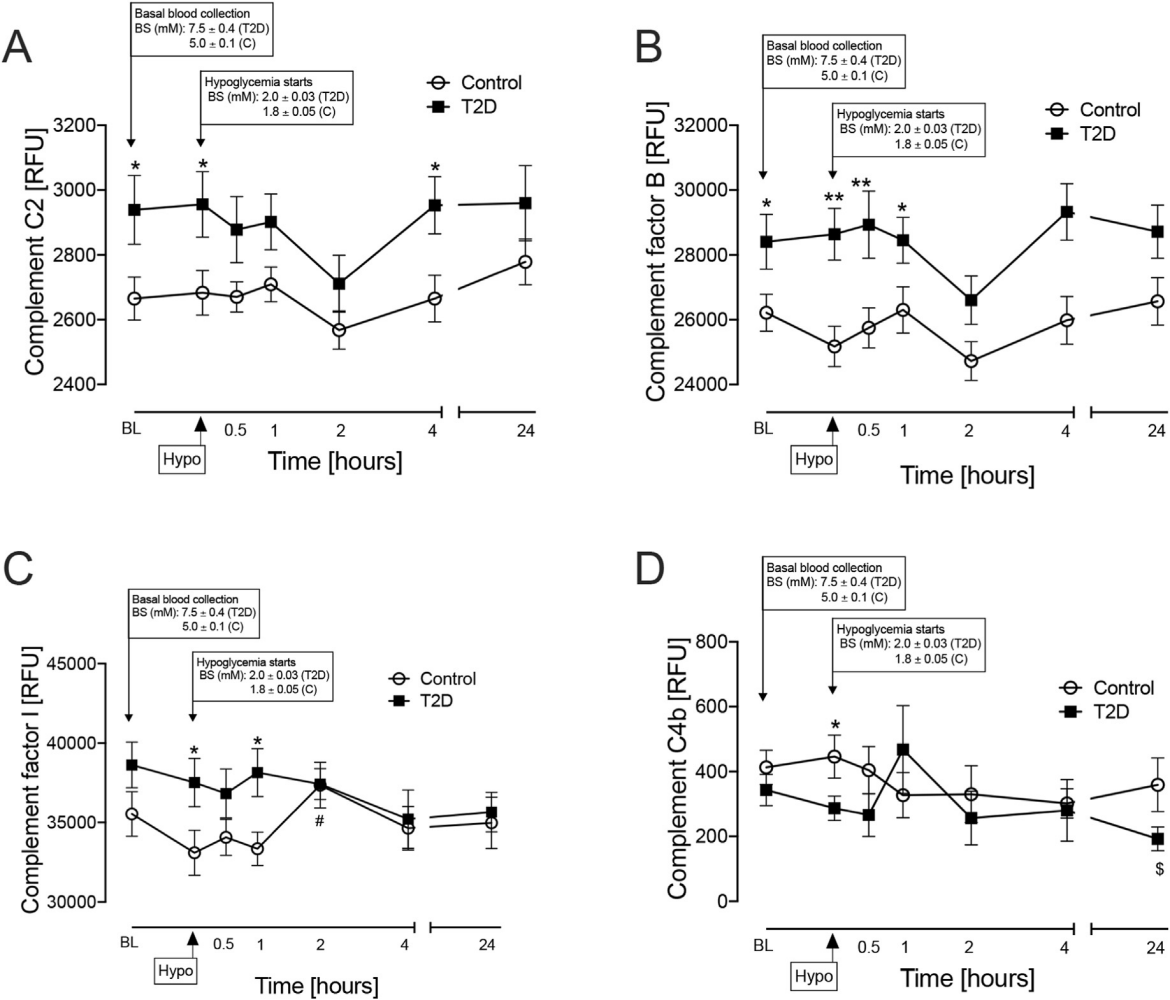
Complement pathway-related proteins that differed between type 2 diabetes. (T2D) and control subjects either at baseline or at the point of hypoglycaemia.Proteomic (Somalogic) analysis of Complement C2 (A), Factor B (B), Factor I (C) and C4b (D). Statistics: (∗p < 0.05, ∗∗p < 0.01, control vs T2D); (# p < 0.05, control hypoglycaemia vs control 2-h post-hypoglycemia); ($p < 0.05 T2D baseline vs T2D 24-h post-hypoglycemia). Controls (white circles), T2D (black squares); RFU, relative fluorescent units.

In the T2D cohort, Complement C2 (p < 0.05) and Factor B (p < 0.05) were elevated at baseline versus controls (Fig. 2, A-B).

At hypoglycaemia, Complement C2 (p < 0.05) and Factor B (p < 0.01) remained elevated, whilst Factor I became elevated (p < 0.05) in the T2D cohort (Fig. 2C); by contrast, Complement C4b became elevated in controls (p < 0.05) (Fig. 2D).

**Fig. 3:**
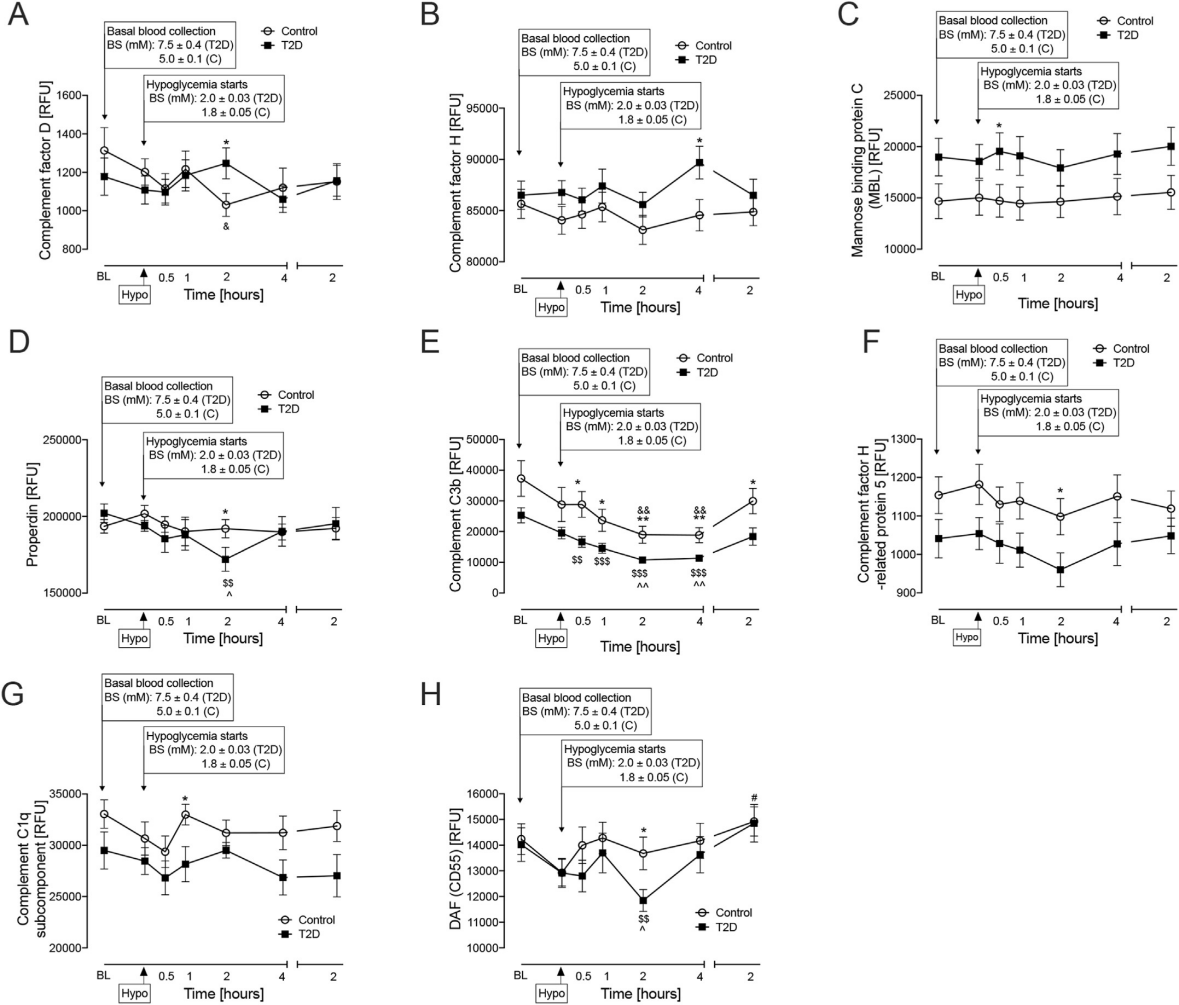
Complement pathway-related proteins that differed between type 2 diabetes (T2D) and control subjects at post-hypoglycemia timepoints. Proteomic (Somalogic) analysis of Factor D (A), Factor H (B), Mannose binding protein C (MBL) (C), Properdin (D), C3b (E), Factor H (F), C1q (G) and Complement decay-accelerating factor (CD55 or DAF) (H). Statistics: (∗p < 0.05, control vs T2D) (& p < 0.05, control baseline vs control 2-h post-hypoglycemia) (# p < 0.05, control hypoglycaemia vs control 2-h post-hypoglycemia) ($$ p < 0.01, $$$ p < 0.001, T2D baseline vs T2D post-hypoglycemia timepoints) (^p < 0.05, ^^p < 0.01, T2D hypoglycaemia vs T2D post-hypoglycemia timepoints). Controls (white circles), T2D (black squares); RFU, relative fluorescent units.

In the post-hypoglycemia follow up period, Complement C2 and Factor B remained elevated in T2D (**Complement C2** at 4-h p < 0.05; **Factor B** at 30mins p < 0.001, 1-h p < 0.05 and 4-h p < 0.01). Factor I was elevated until the 2-h timepoint in T2D (1-h, p < 0.05), at which and thereafter levels were similar to controls (Fig. 2, A-C). Factor D was elevated only at the 2-h timepoint in T2D (p < 0.05), whilst Factor H and mannose-binding protein C showed elevations in T2D versus controls throughout the time course (**Factor H** 4-h, p < 0.05; **mannose-binding protein C** 30-min, p < 0.05) (Fig. 3, A-C); properdin, complement C3b, Factor H-related protein 5, complement C1q and Complement decay-accelerating factor (CD55 or DAF) showed elevations in controls versus T2D at varying post-hypoglycemia timepoints (**Properdin** 2-h, p < 0.05; **complement C3b** [30-min, p < 0.05; 1-h, p < 0.05; 2-h, p < 0.01; 4-h, p < 0.01; 24-h, p < 0.05]; **Factor H-related protein 5**, 2-h, p < 0.05; **complement C1q**, 1-h: p < 0.05; **DAF**, 2-h: p < 0.05) (Fig. 3, D-H).

Supplementary Table 1A details the p-value, % difference, fold difference and standard deviation (SD) for the proteins that differed between T2D and controls.

### Within cohort changes at hypoglycaemia and post-hypoglycemia

There were no significant differences for any of the complement pathway-related proteins at the point of hypoglycaemia versus baseline in either the T2D or control group.

#### Post-hypoglycemia changes within group for controls only

Factor I increased at 2-h post-hypoglycemia versus hypoglycemia (p < 0.05) (Fig. 2C).

Factor D decreased at 2-h versus baseline (p < 0.05) in controls only (p < 0.05) (Fig. 3A).

**Fig. 4:**
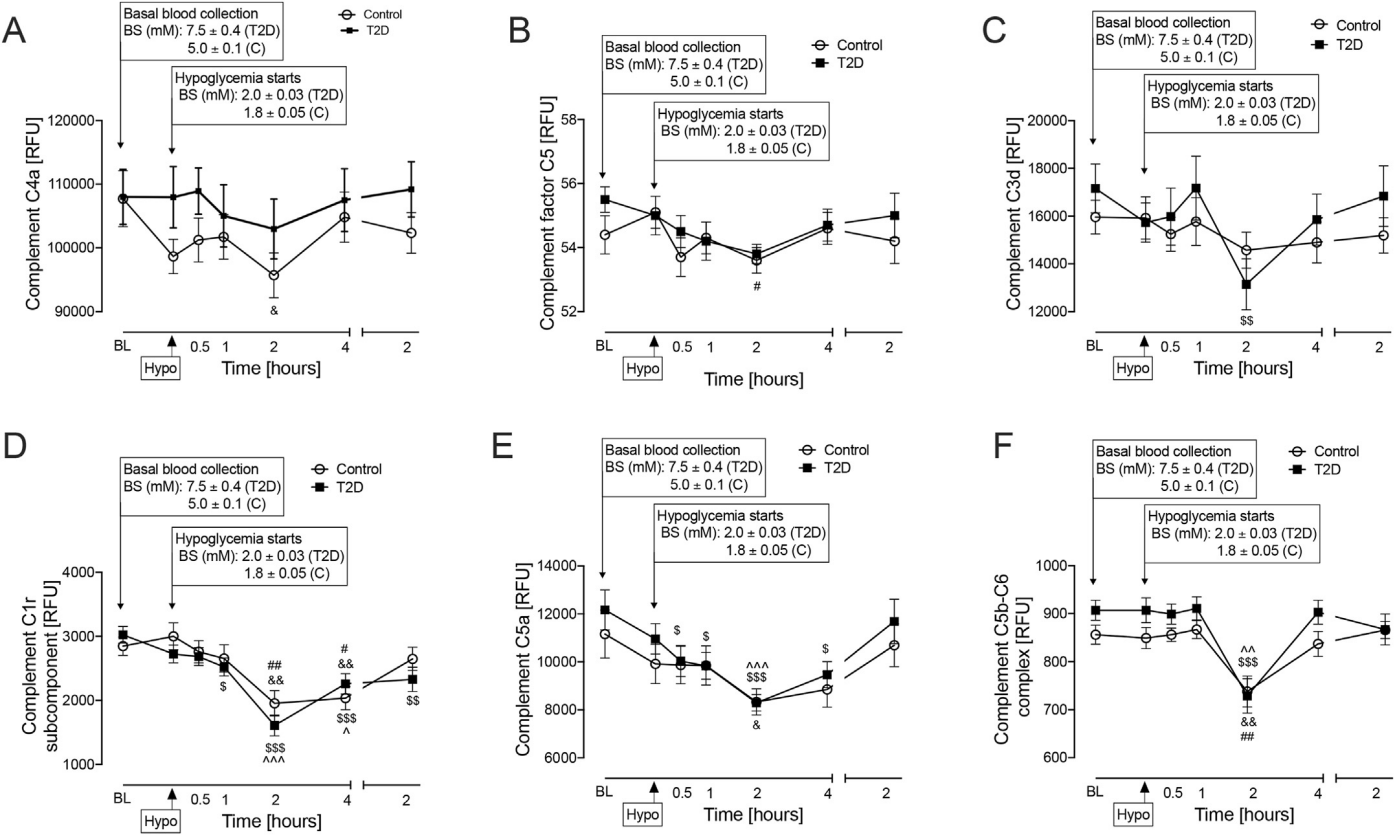
Complement pathway-related proteins that differed within group for type 2 diabetes (T2D) or control subjects at the post-hypoglycemia timepoints. Proteomic (Somalogic) analysis of Complement C4a (A), C5 (B), C3d (C), C1r (D), C5a (E) and C5b-C6 complex (F). Statistics: (& p < 0.05, && p < 0.01, control baseline vs control post-hypoglycemia timepoints) (# p < 0.05, ## p < 0.01 control hypoglycaemia vs control post-hypoglycemia timepoints) ($ p < 0.05. $$ p < 0.01, $$$ p < 0.001 T2D baseline vs T2D post-hypoglycemia timepoints) (^p < 0.05, ^^p < 0.01,^^^p < 0.001, T2D hypoglycaemia vs T2D post-hypoglycemia timepoints); controls (white circles), T2D (black squares). RFU, relative fluorescent units.

Complement C4a decreased in controls only at 2-h post-hypoglycemia versus baseline (p < 0.05) (Fig. 4A).

Complement C5 decreased in controls only at 2-h post-hypoglycemia versus baseline (p < 0.05) (Fig. 4B).

#### Post-hypoglycemia changes within group for T2D only

Properdin decreased at 2-h post-hypoglycemia versus baseline in T2D only (p < 0.01) (Fig. 3D).

DAF decreased at the 2-h post-hypoglycemia timepoint versus baseline in T2D only (p < 0.01) (Fig. 3H).

Complement C3d decreased at the 2-h post-hypoglycemia timepoint versus baseline in T2D only (p < 0.01) (Fig. 4C).

#### Post-hypoglycemia changes within group for both controls and T2D

Complement C3b decreased from 30-min to 4-h post-hypoglycemia in T2D versus baseline (30-min, p < 0.01; 1-h, p < 0.001; 2-h, p < 0.001; 4-h, p < 0.001); a decrease was also seen in controls from 2 to 4 h post-hypoglycemia (2-h, p < 0.001; 4-h: p < 0.001) (Fig. 3E).

Complement C1r showed a decrease from 1-h to 24-h, with the most significant decrease at 2-h, post-hypoglycemia versus baseline in T2D (1-h, p < 0.05; 2-h, p < 0.001; 4-h, p < 0.001; 24-h, p < 0.01) likewise, in controls, there was a decrease from baseline between 2-h and 4-h post-hypoglycemia (2-h, p < 0.001; 4-h, p = 0.001) (Fig. 4D).

Complement C5a decreased in T2D from 30-min to 4-h versus baseline, with the most significant decrease at 2-h, post-hypoglycemia (30-min, p < 0.05; 1-h, p < 0.05; 2-h, p < 0.001; 4-h, p < 0.05); a decrease was also seen in controls at 2-h post-hypoglycemia (p = 0.05) (Fig. 4E).

Complement C5b-C6 complex decreased in both T2D (p < 0.001) and controls (p < 0.01) at the 2-h post-hypoglycemia timepoint versus baseline (Fig. 4F).

Supplementary Table 1B details the p-value, % change, fold change and SD for the proteins that showed ‘within cohort’ changes over the study time course.

There were no differences either between T2D and control groups or within T2D and control groups throughout the timecourse for the following complement pathway-related proteins: Complement C3, iC3b, C3a, C8a, MASP1 and CCL2 (Supplementary Fig. 1, A-F).

**Fig. 5:**
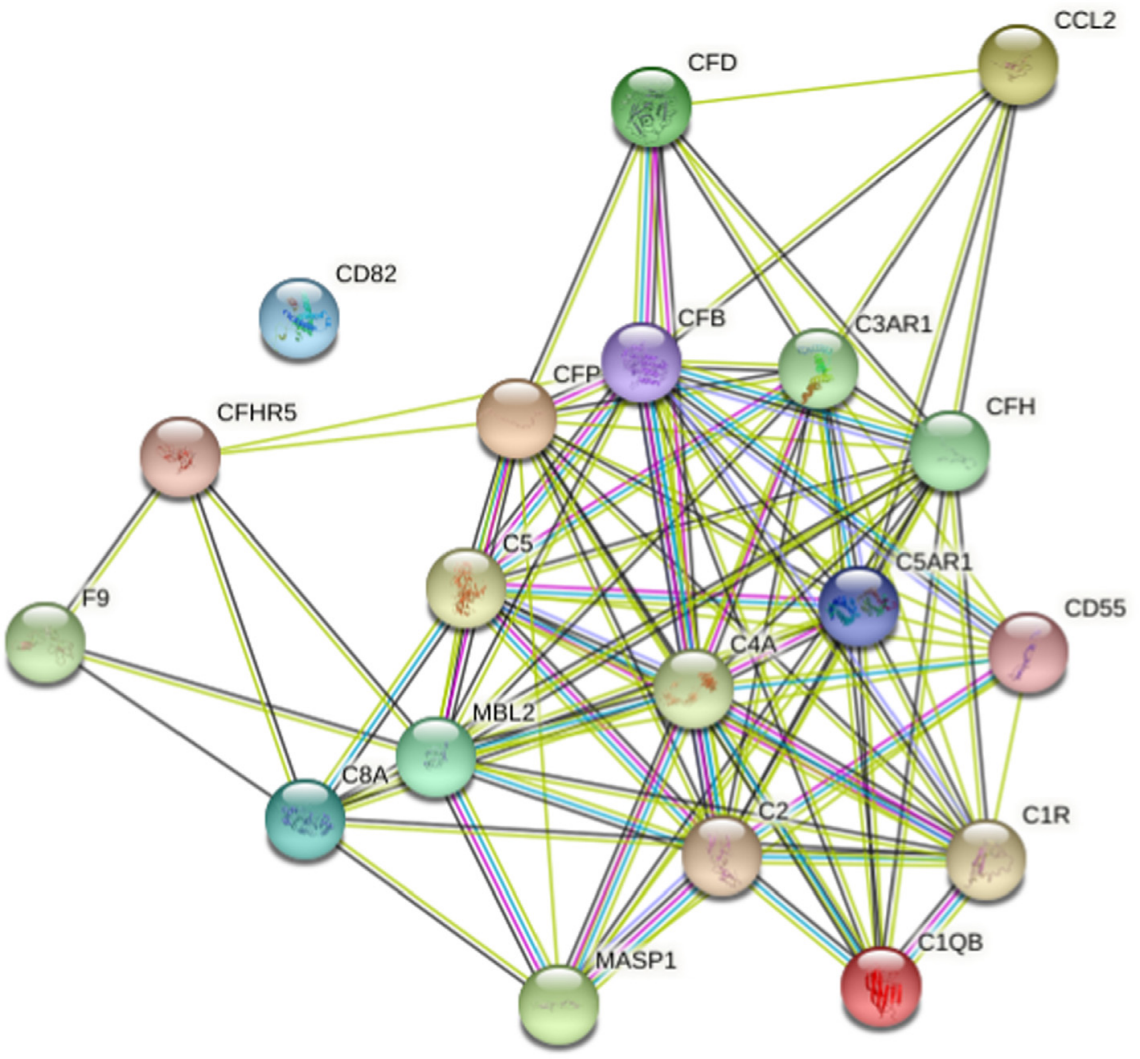
STRING interaction network. STRING version 11.0 interaction network showing the interactions of complement pathway-related proteins. The STRING 11.0 (Search Tool for the Retrieval of Interacting Genes) was used to visualize the known and predicted Protein-Protein Interactions for proteins identified by SOMAscan assay in plasma of T2D versus control subjects (https://string-db.org/).

STRING analysis revealed the close linkage between complement pathway-related proteins (Fig. 5).

### Correlation between age, BMI and inflammatory markers with complement pathway-related proteins that differed at baseline between T2D and controls

No correlation was found between age or BMI and basal levels of Complement C2 and Factor B, the two complement pathway-related proteins that differed at baseline between T2D and control subjects.

Interleukin-1 correlated with baseline C2 in T2D only (r = 0.42, p = 0.046) and correlated with Factor B in controls only (r = 0.43, p = 0.039). Interferon-γ did not correlate with baseline levels of C2 and Factor B in either T2D or controls.

### Partial correlation and Granger causality analysis

To determine the degree of association between proteins from the complement and inflammatory pathways, a partial correlation analysis was performed. Whilst many proteins within either the complement or the inflammatory pathways showed close association, that was to be anticipated. Close association of proteins between pathways, defined as a p-value <0.05, was limited to 12 pairs of proteins (Table 1).

**Table 1:** Partial correlation analysis depicting proteins from the complement cascade and inflammatory pathways that were closely associated.

Protein	Protein ID	Category	Protein	Protein ID	Category	p-value
CFHR5	Q9BXR6	complement	FGF8	P55075	inflammatory	0.005
C1q	P02745...P02746..P02747	complement	CXCL10	P02778	inflammatory	0.008
NCR1	O76036	complement	CXCL10	P02778	inflammatory	0.011
CCL2	P13500	complement	CXCL10	P02778	inflammatory	0.033
C3	P01024	complement	IL12A	P29459...P29460.	inflammatory	0.034
NCR1	O76036	complement	CCL19	Q99731	inflammatory	0.035
CFHR5	Q9BXR6	complement	TNFa	P01375	inflammatory	0.036
CCL2	P13500	complement	IL10	P22301	inflammatory	0.038
CCL2	P13500	complement	FGF8	P55075	inflammatory	0.038
C1r	P00736	complement	TBK1	Q9UHD2	inflammatory	0.041
C4A	P0C0L4..P0C0L5 ….P0C0L4..P0C0L5	complement	IL10	P22301	inflammatory	0.042
C1q	P02745...P02746..P02747	complement	IL1A	P01583	inflammatory	0.050

CFHR5, complement factor H-related protein 5; C1q, complement component 1q; NCR1, natural cytotoxicity triggering receptor 1; CCL2, C–C motif chemokine ligand 2; C3, complement component 3; C1r, complement component 1r; C1q, complement component 1q; C4A, complement component 4A; FGF8, fibroblast growth factor 8; CXCL10, C-X-C motif chemokine ligand 10; IL12A, interleukin 12 alpha; CCL19, C–C motif chemokine ligand 19; TNFa, tumour necrosis factor alpha; IL10, interleukin 10; TBK1, TANK-binding kinase 1; IL1A, interleukin 1 alpha.

In order to test whether changes in complement pathway proteins were prompted by changes in inflammatory pathway proteins or vice versa, the Granger causality analysis was performed on the inflammatory and complement proteins. This analysis tests whether a variable of interest can be predicted based on the measurement of another variable from a previous timepoint.

We have previously shown that a number of inflammatory proteins are affected by hypoglycaemia [34] and these include C-X-C motif chemokine ligand 10 (CXCL10), interleukin 1 alpha (IL-1A), interleukin 10 (IL-10), interleukin 12 alpha (IL-12A), C–C motif chemokine ligand 19 (CCL19), tumour necrosis factor alpha (TNF-alpha) and TANK-binding kinase 1 (TBK1) and therefore these specific proteins were correlated with the complement proteins.

Protein pairs involving one complement and one inflammatory protein revealed by this lagged analysis are shown in Table 2. It is apparent that inflammatory proteins drive complement proteins more frequently in T2D than in controls, likely because the baseline levels of inflammatory proteins are elevated in T2D. By contrast, in controls, it is the complement proteins that more often drive the inflammatory proteins, whereas this lagged association is very rare in T2D.

**Table 2:** Granger causality analysis indicating the proteins from either the complement cascade or the inflammatory pathway that could be predicted based on the measurement of a protein from a previous timepoint from the opposite pathway.

A. Inflammation driving complement					
CONTROLS			T2D		
Protein pair	lagged comparison	p-value	Protein pair	lagged comparison	p-value
IL5-FactorB	P05113–P00751	0.016	S100A9-MBL2	P06702–P11226	0.001
RPS6KA5-C5A	O75582–P01031	0.018	S100A9-MASP1	P06702–P48740	0.013
IL1A-C3	P01583–P01024.1	0.018	IL1A-C4A	P01583-HCE001796	0.013
HMGB1-NCR1	P09429–O76036	0.026	IL10-MBL2	P22301–P11226	0.014
IL1A-NCR1	P01583–O76036	0.032	IL5-CD55 (DAF)	P05113–P08174	0.016
IL12A-C2	P29459⋯P29460.-P06681	0.047	SIGLEC1-C3	Q9BZZ2-P01024.3	0.018
HMGB1-C8A	P09429–P07357.P07358.P07360.	0.048	HMGB1-CCL7	P09429–P80098	0.025
IL12A-CD55 (DAF)	P29459⋯P29460.-P08174	0.049	S100A9-CD55 (DAF)	P06702–P08174	0.026
			S100A9-NCR1	P06702–O76036	0.028
			PTGS2-NCR1	P35354–O76036	0.029
			PRKCZ-Properdin	Q05513-P27918	0.033
			IL12-C4A	P29459⋯P29460.-HCE001796	0.036
			IL34-CLU	Q6ZMJ4-P10909	0.036
			TNFa-CCL8	P01375–P80075	0.039
			IL5-MBL2	P05113–P11226	0.042
			RPS6KA5-CD55 (DAF)	O75582–P08174	0.045
			HMGB1-CFHR5	P09429-Q9BXR6	0.045
			CCL19-C3	Q99731-P01024.2	0.045
			IL10-MASP1	P22301–P48740	0.046
			IL1A-C3	P01583–P01024.4	0.047
			IL34-Properdin	Q6ZMJ4-P27918	0.049
			IL5-MASP1	P05113–P48740	0.050

B. Complement driving Inflammation					
CONTROLS			T2D		
Protein pair	lagged comparison	p-value	Protein pair	lagged comparison	p-value
FactorI-HMGB1	P05156–P09429	0.006	MBL2-RPS6KA5	P11226–O75582	0.016
FactorI-RPS6KA5	P05156–O75582	0.013	C5-IL34	HCE004152-Q6ZMJ4	0.023
C1q-AZU1	P02745⋯P02746..P02747–P20160	0.014			
C3-TNFa	P01024–P01375	0.014			
C5A-HMGB1	P01031.P13671.-P09429	0.019			
C8A-TNFa	P07357.P07358.P07360.-P01375	0.020			
C4-IL1A	HCE001796-P01583	0.020			
C3-IL10	P01024–P22301	0.022			
C1q-TNFa	P02745⋯P02746..P02747–P01375	0.027			
C1r-CD40LG	P00736–P29965	0.029			
C3-IFNg	P01024.4-P01579	0.029			
CCL2-HMGB1	P13500–P09429	0.037			
C1r-HMGB1	P00736–P09429	0.037			
CCL8-HMGB1	P80075–P09429	0.038			
CLU-CD40LG	P10909–P29965	0.047			

IL5, interleukin 5; Factor B, complement factor B; RPS6KA5, ribosomal protein S6 kinase A5; C5A, complement component 5A; IL1A, interleukin 1 alpha; C3, complement component 3; HMGB1, high mobility group box 1 protein; NCR1, natural cytotoxicity triggering receptor 1; IL12A, interleukin 12 alpha; C2, complement component 2; C8A, complement component 8A; CD55(DAF), cluster of differentiation protein 55 (decay-accelerating factor); S100A9, s100 calcium-binding protein A9; MBL2, mannose-binding protein C; MASP1, mannan-binding lectin serine protease 1; C4A, complement component 4A; IL10, interleukin 10; IL5, interleukin 5; SIGLEC1, sialoadhesin (sialic acid-binding Ig-like lectin 1); CCL7, C–C motif chemokine ligand 7; PTGS2, prostaglandin G/H synthase 2; PRKCZ, protein kinase C zeta type; IL12, interleukin 12; IL34, interleukin 34; CLU, clusterin; TNFa, tumour necrosis factor alpha; CCL8, C–C motif chemokine ligand 8; CFHR5, complement factor H-related protein 5; CCL19, C–C motif chemokine ligand 19; Factor I, complement factor I;; C1q, complement component 1q; AZU1, azurocidin; C4, complement component 4; IL10, interleukin 10; C1r, complement component 1r; CD40LG, cluster of differentiation 40 ligand; IFNg, interferon gamma; CCL2, C–C motif chemokine ligand 2; C5, complement component 5.

## Discussion

The results of this study show an elevation of Complement C2 and Factor B at baseline in T2D versus control subjects. Upon induction of hypoglycaemia, C2 and Factor B remained elevated, whilst Factor I became elevated in T2D and C4b became elevated in controls. Perturbations of complement pathway-related proteins occurred largely around the 2-h post-hypoglycemia timepoint and had, in almost all cases, returned to baseline at 24-h, demonstrating an acute but transient response.

The elevated complement C2 and Factor B at baseline in T2D subjects without any diabetes-related complications, and that remained consistently higher at all time points of the hypoglycemic challenge, are novel observations. Complement C2 is important in the lectin and classical pathways of complement activation and is important in the formation of C3-convertase and C5-convertase activation [24]; however, its upregulation in T2D has not been described before and theoretically may indicate that the complement system is primed for activation in diabetes. This is reflected in the observation that the complement pathway shows increased activation in T2D [35]. Interestingly, Factor B, a complement factor in the alternative pathway, is also important in the formation of C3-convertase and C5-convertase activation [24]. Upregulation of both C2 and Factor B seen at baseline in T2D may be responsible for the well described increased levels of C3 and C3 activator in diabetes that are related to glucose intolerance and elevated fasting plasma glucose [35]. From the Granger causality analysis, it can be seen that inflammation was driving complement, and all the more so in the T2D cohort, where underlying chronic inflammation is well recognised and where these factors may be primed to respond. However, it is evident that complement changes were also driving inflammation, suggesting that a vicious circle of complement and inflammatory marker activation may result. That there is upregulation of complement C2 and Factor B may indicate that these are driven by metabolic factors [35] rather than inflammation. C2 and Factor B are closely linked, as they are encoded by single loci in the Class III region of the major histocompatibility complex on the short arm of chromosome 6 [36]. C2 in the classical pathway is the functional homologue of Factor B in the alternative pathway.

In the classical pathway, C2 associates with activated C4 (C4b) and is cleaved by activated C1 into 2 fragments, C2a and C2b. C2a combines with factor C4b to generate C3- and C5-convertases In the alternative pathway, C3-convertase is formed from C3 and Factor B: Factor B associates with C3b and is cleaved by Factor D into Ba and Bb, and the association of C3b and Bb is the alternative pathway C3-convertase [36] (Fig. 1).

C2 and Factor B are synthesized in the liver, the source of the majority of circulating protein, and in monocyte/macrophage lineage cells. Their expression can be induced by inflammatory proteins, such as interferon-γ and interleukin-1 [37], the latter correlating with C2 and Factor B here, and likely accounting for the increased basal levels in T2D versus controls through the increased basal levels of the inflammatory proteins seen in T2D [34].

In accord with our findings, baseline Factor B has previously been reported to be elevated in serum of Asian patients with T2D [38] and its expression in adipose tissue correlates with fasting glucose and circulatory lipids [39,40]. Elevated levels of circulatory Factor B are reported to increase the risk of endothelial dysfunction [41] and coronary heart disease [42]. Despite its homology to Factor B, little has been reported about the role of C2 in disease.

Acquired causes of low complement levels include autoimmune disorders, such as systemic lupus erythematosis, several forms of glomerulonephritis, vasculitides and autoimmune pancreatitis; the common underlying mechanism is accelerated consumption of proteins by immune complexes [43,44]. This mechanism is the likely cause of the transient fall in complement pathway-related proteins seen in this study at 2-h following the hypoglycemic insult.

This study benefited from the inclusion of normal controls to define what a normal physiological response may be to hypoglycemia, with the caveat that non iatrogenic hypoglycemia is a rare and pathological event in non-diabetic individuals. However, it highlights the impact of the pathophysiological changes that T2D may have even when of relatively short duration. The complement protein changes seen in response to hypoglycemia were mainly seen at 2-h in both controls and T2D, and often showed a decrease in complement protein levels that was transient, reverting to basal levels within 2-h. In the controls, complement factors C3, C4, D, C5, C5a, C5b-C6 complex and C1r all significantly decreased at 2 h compared to baseline. In T2D, complement factors C3, C3b, C3a desArg, C3d, C5, C5a, C5b-C6 complex C1r and CD55. This suggests that in controls and in T2D that both the classical and alternative complement pathways were being activated as shown in Fig. 1. Complement levels can fall to very low levels within a few hours due to the development of immune complexes so that the fall in complement here may be a reflection of activation; consumption of complement in response to the physiological stress response and reduction of complement levels have been reported in acute pancreatitis with the levels of C3 and C4 being sensitive and accurate to the severity of the pancreatitis [45], whilst a fall in C3 and its reversibility was predictive in renal allograft rejection and recovery [46].

The drivers of the change in complement response to hypoglycemia differed between controls and T2D. Beyond its role in pathogen elimination, the complement system is a recognised mediator of the inflammatory response [22,23]. While partial correlation analysis infers considerable interaction between the complement and inflammatory pathways, as evidenced by the string pathway analysis, the Granger causality analysis revealed that in controls hypoglycemia induced a response in complement that then affected the inflammatory proteins, whilst, conversely, in T2D it was largely the inflammatory proteins that drove the complement cascade. That inflammatory proteins are the drivers in T2D may be explained by their upregulation even in the basal state in T2D [34]. Hypothetically, chronic low-grade inflammation may prime the complement pathway proteins; when a hypoglycemic episode occurs, this may initiate an inflammatory response/cascade that may be prolonged beyond the transient complement changes and could thereby contribute to a cardiovascular event. Others have shown that the impact of a hypoglycemic effect may still be evident after seven days and, whilst complement C3 was not altered at the time of the hypoglycemic event, it became elevated at seven days suggesting that the inflammatory cascade had continued for this period of time [47]. This accords with the well described vicious cycle of the inflammatory process leading to complement activation leading to further inflammatory stimulation [48].

This study has a number of limitations. Firstly, the relatively small subject numbers in this intensive study protocol may not have identified additional alterations in complement proteins though a lack of power; as this was a pilot study, larger appropriately powered confirmatory studies should be undertaken. Secondly, we report the complement protein levels and not their activation, and functional studies are needed to confirm these findings particularly with regards to the link between inflammation and complement levels. Thirdly, this was a homogenous Caucasian population and its applicability to other ethnicities needs confirmation.

In conclusion, baseline elevations in C2 and Factor B indicate upregulation of the complement pathway in T2D and changes in complement pathway-related protein levels in response to hypoglycemia suggest both intrinsic and alternative pathway activation at 2-h that appear driven by the underlying inflammation in T2D and that could contribute to a cardiovascular event. It is, however, important to note that this was a pilot study and further larger, appropriately powered studies are needed to confirm and extend these findings.

## Author contributions

ASMM, MN and AEB analyzed the data and wrote the manuscript. ID performed the statistical analysis. AA-Q performed the clinical studies. TS supervised clinical studies and edited the manuscript. SLA contributed to study design, data interpretation and the writing of the manuscript. All authors reviewed and approved the final version of the manuscript. Alexandra E Butler is the guarantor of this work.

## Ethics approval and consent to participate

The Newcastle & North Tyneside Ethics committee approved this study that was conducted according to the Declaration of Helsinki. All study participants signed an informed consent form prior to participation.

## Consent for publication

All authors gave their consent for publication.

## Availability of data and materials

All the data for this study will be made available upon reasonable request to the corresponding author.

## Funding

No funding was received to perform this study.

## Declaration of competing interest

The authors declare that they have no known competing financial interests or personal relationships that could have appeared to influence the work reported in this paper.

The authors declare the following financial interests/personal relationships which may be considered as potential competing interests:

## Figures and Tables

**Figure:**
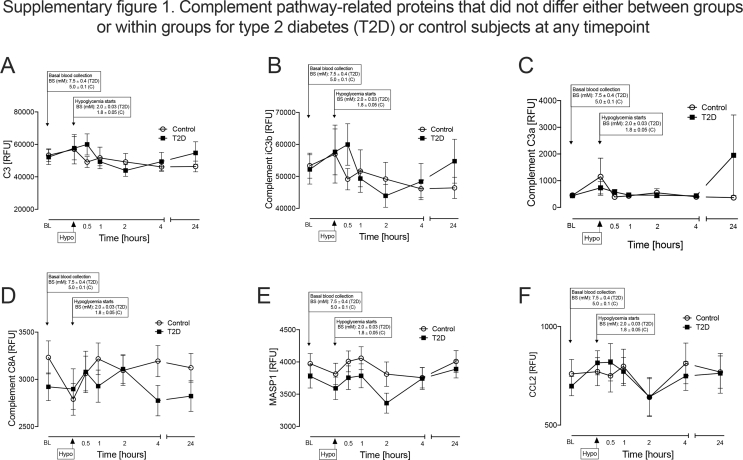
figs1Complement pathway-related proteins that did not differ either between groups or within groups for type 2 diabetes (T2D) or control subjects at any timepoint. Proteomic (Somalogic) analysis of Complement C3 (A), iC3b (B), C3a (C), C8A (D), MASP1 (E) and CCL2 (F). Controls (white circles), T2D (black squares); RFU, relative fluorescent units.

## References

[1] Diabetes Study G., Gerstein H.C., Miller M.E., Byington R.P., Goff D.C., Bigger J.T., Action to Control Cardiovascular Risk (2008). Effects of intensive glucose lowering in type 2 diabetes. N Engl J Med.

[2] Group A.C., Patel A., MacMahon S., Chalmers J., Neal B., Billot L. (2008). Intensive blood glucose control and vascular outcomes in patients with type 2 diabetes. N Engl J Med.

[3] Miller M.E., Bonds D.E., Gerstein H.C., Seaquist E.R., Bergenstal R.M., Calles-Escandon J. (2010). The effects of baseline characteristics, glycaemia treatment approach, and glycated haemoglobin concentration on the risk of severe hypoglycaemia: post hoc epidemiological analysis of the ACCORD study. BMJ.

[4] Bonds D.E., Miller M.E., Bergenstal R.M., Buse J.B., Byington R.P., Cutler J.A. (2010). The association between symptomatic, severe hypoglycaemia and mortality in type 2 diabetes: retrospective epidemiological analysis of the ACCORD study. BMJ.

[5] Investigators O.T., Mellbin L.G., Ryden L., Riddle M.C., Probstfield J., Rosenstock J. (2013). Does hypoglycaemia increase the risk of cardiovascular events? A report from the ORIGIN trial. Eur Heart J.

[6] Investigators N.-S.S., Finfer S., Chittock D.R., Su S.Y., Blair D., Foster D. (2009). Intensive versus conventional glucose control in critically ill patients. N Engl J Med.

[7] Bedenis R., Price A.H., Robertson C.M., Morling J.R., Frier B.M., Strachan M.W. (2014). Association between severe hypoglycemia, adverse macrovascular events, and inflammation in the Edinburgh Type 2 Diabetes Study. Diabetes Care.

[8] Kosiborod M., Inzucchi S.E., Goyal A., Krumholz H.M., Masoudi F.A., Xiao L. (2009). Relationship between spontaneous and iatrogenic hypoglycemia and mortality in patients hospitalized with acute myocardial infarction. J Am Med Assoc.

[9] Goto A., Arah O.A., Goto M., Terauchi Y., Noda M. (2013). Severe hypoglycaemia and cardiovascular disease: systematic review and meta-analysis with bias analysis. BMJ.

[10] Davis S.N., Duckworth W., Emanuele N., Hayward R.A., Wiitala W.L., Thottapurathu L. (2019). Effects of severe hypoglycemia on cardiovascular outcomes and death in the veterans Affairs diabetes trial. Diabetes Care.

[11] Group B.D.S., Frye R.L., August P., Brooks M.M., Hardison R.M., Kelsey S.F. (2009). A randomized trial of therapies for type 2 diabetes and coronary artery disease. N Engl J Med.

[12] Boden G., Vaidyula V.R., Homko C., Cheung P., Rao A.K. (2007). Circulating tissue factor procoagulant activity and thrombin generation in patients with type 2 diabetes: effects of insulin and glucose. J Clin Endocrinol Metab.

[13] Seljeflot I., Larsen J.R., Dahl-Jorgensen K., Hanssen K.F., Arnesen H. (2006). Fibrinolytic activity is highly influenced by long-term glycemic control in Type 1 diabetic patients. J Thromb Haemostasis.

[14] Colwell J.A., Nesto R.W. (2003). The platelet in diabetes: focus on prevention of ischemic events. Diabetes Care.

[15] Dalsgaard-Nielsen J., Madsbad S., Hilsted J. (1982). Changes in platelet function, blood coagulation and fibrinolysis during insulin-induced hypoglycaemia in juvenile diabetics and normal subjects. Thromb Haemostasis.

[16] Trovati M., Anfossi G., Cavalot F., Vitali S., Massucco P., Mularoni E. (1986). Studies on mechanisms involved in hypoglycemia-induced platelet activation. Diabetes.

[17] Hutton R.A., Mikhailidis D., Dormandy K.M., Ginsburg J. (1979). Platelet aggregation studies during transient hypoglycaemia: a potential method for evaluating platelet function. J Clin Pathol.

[18] Razavi Nematollahi L., Kitabchi A.E., Stentz F.B., Wan J.Y., Larijani B.A., Tehrani M.M. (2009). Proinflammatory cytokines in response to insulin-induced hypoglycemic stress in healthy subjects. Metabolism.

[19] Wright R.J., Newby D.E., Stirling D., Ludlam C.A., Macdonald I.A., Frier B.M. (2010). Effects of acute insulin-induced hypoglycemia on indices of inflammation: putative mechanism for aggravating vascular disease in diabetes. Diabetes Care.

[20] Gogitidze Joy N., Hedrington M.S., Briscoe V.J., Tate D.B., Ertl A.C., Davis S.N. (2010). Effects of acute hypoglycemia on inflammatory and pro-atherothrombotic biomarkers in individuals with type 1 diabetes and healthy individuals. Diabetes Care.

[21] Dandona P., Chaudhuri A., Dhindsa S. (2010). Proinflammatory and prothrombotic effects of hypoglycemia. Diabetes Care.

[22] Leslie M. (2012). Immunology. The new view of complement. Science.

[23] Ricklin D., Hajishengallis G., Yang K., Lambris J.D. (2010). Complement: a key system for immune surveillance and homeostasis. Nat Immunol.

[24] Shim K., Begum R., Yang C., Wang H. (2020). Complement activation in obesity, insulin resistance, and type 2 diabetes mellitus. World J Diabetes.

[25] Rasmussen K.L., Nordestgaard B.G., Nielsen S.F. (2018). Complement C3 and risk of diabetic microvascular disease: a cohort study of 95202 individuals from the general population. Clin Chem.

[26] Fujita T., Hemmi S., Kajiwara M., Yabuki M., Fuke Y., Satomura A. (2013). Complement-mediated chronic inflammation is associated with diabetic microvascular complication. Diabetes Metab Res Rev.

[27] Mellbin L.G., Bjerre M., Thiel S., Hansen T.K. (2012). Complement activation and prognosis in patients with type 2 diabetes and myocardial infarction: a report from the DIGAMI 2 trial. Diabetes Care.

[28] Moin A.S.M., Al-Qaissi A., Sathyapalan T., Atkin S.L., Butler A.E. (2020). Hypoglycaemia in type 2 diabetes exacerbates amyloid-related proteins associated with dementia. Diabetes Obes Metabol.

[29] Al-Qaissi A., Papageorgiou M., Deshmukh H., Madden L.A., Rigby A., Kilpatrick E.S. (2019). Effects of acute insulin-induced hypoglycaemia on endothelial microparticles in adults with and without type 2 diabetes. Diabetes Obes Metabol.

[30] Hepburn D.A., Frier B.M. (1991). Hypoglycemia unawareness in patients with insulin-treated diabetes-mellitus. Saudi Med J.

[31] Kraemer S., Vaught J.D., Bock C., Gold L., Katilius E., Keeney T.R. (2011). From SOMAmer-based biomarker discovery to diagnostic and clinical applications: a SOMAmer-based, streamlined multiplex proteomic assay. PLoS One.

[32] Suhre K., Arnold M., Bhagwat A.M., Cotton R.J., Engelke R., Raffler J. (2017). Connecting genetic risk to disease end points through the human blood plasma proteome. Nat Commun.

[33] Birkett M.A., Day S.J. (1994). Internal pilot studies for estimating sample size. Stat Med.

[34] Kahal H., Halama A., Aburima A., Bhagwat A.M., Butler A.E., Graumann J. (2020). Effect of induced hypoglycemia on inflammation and oxidative stress in type 2 diabetes and control subjects. Sci Rep.

[35] McMillan D.E. (1980). Elevation of complement components in diabetes mellitus. Diabete Metab.

[36] Campbell R.D. (1987). The molecular genetics and polymorphism of C2 and factor B. Br Med Bull.

[37] Strunk R.C., Cole F.S., Perlmutter D.H., Colten H.R. (1985). gamma-Interferon increases expression of class III complement genes C2 and factor B in human monocytes and in murine fibroblasts transfected with human C2 and factor B genes. J Biol Chem.

[38] Somani R., Richardson V.R., Standeven K.F., Grant P.J., Carter A.M. (2012). Elevated properdin and enhanced complement activation in first-degree relatives of South Asian subjects with type 2 diabetes. Diabetes Care.

[39] Coan P.M., Barrier M., Alfazema N., Carter R.N., Marion de Proce S., Dopico X.C. (2017). Complement factor B is a determinant of both metabolic and cardiovascular features of metabolic syndrome. Hypertension.

[40] Moreno-Navarrete J.M., Martinez-Barricarte R., Catalan V., Sabater M., Gomez-Ambrosi J., Ortega F.J. (2010). Complement factor H is expressed in adipose tissue in association with insulin resistance. Diabetes.

[41] Hertle E., Arts I.C., van der Kallen C.J., Feskens E.J., Schalkwijk C.G., Stehouwer C.D. (2016). The alternative complement pathway is longitudinally associated with adverse cardiovascular outcomes. The CODAM study. Thromb Haemostasis.

[42] Donahue M.P., Rose K., Hochstrasser D., Vonderscher J., Grass P., Chibout S.D. (2006). Discovery of proteins related to coronary artery disease using industrial-scale proteomics analysis of pooled plasma. Am Heart J.

[43] Chimenti M.S., Ballanti E., Triggianese P., Perricone R. (2015). Vasculitides and the complement system: a comprehensive review. Clin Rev Allergy Immunol.

[44] Ramos-Casals M., Campoamor M.T., Chamorro A., Salvador G., Segura S., Botero J.C. (2004). Hypocomplementemia in systemic lupus erythematosus and primary antiphospholipid syndrome: prevalence and clinical significance in 667 patients. Lupus.

[45] Zhang L., Qiao Z., Feng H., Shen J. (2020). The early predictive role of complement C3 and C4 in patients with acute pancreatitis. J Clin Lab Anal.

[46] Kumar P., Kodlin D., Marks C., Leech S.H. (1980). Predictive value of serum complement (C3) in renal allograft rejection. Br J Surg.

[47] Chow E., Iqbal A., Walkinshaw E., Phoenix F., Macdonald I.A., Storey R.F. (2018). Prolonged prothrombotic effects of antecedent hypoglycemia in individuals with type 2 diabetes. Diabetes Care.

[48] Ricklin D., Lambris J.D. (2013). Complement in immune and inflammatory disorders: pathophysiological mechanisms. J Immunol.

