# Supplementary material for: Hypoglycemia-induced changes in complement pathways in type 2 diabetes
Source: Atheroscler Plus. 2021 Nov 18;46:35–45. doi: 10.1016/j.athplu.2021.11.002 (PMC9833243; doi:10.1016/j.athplu.2021.11.002)
Supplement: Multimedia component 1 [file mmc1.docx]

**Supplementary table 1.** Complement proteins that differed between T2D and Controls (A) and those proteins that showed ‘within cohort’ changes over the study time course (B).

| **A. Differences between T2D and Controls** | |  |  |  |  |  |  |
| --- | --- | --- | --- | --- | --- | --- | --- |
| **BASELINE (BL)** | **T2D vs Control** | **p-value** | **% difference at BL** | **Fold difference at BL** | **Standard deviation (SD)** | **Direction** |  |
|  | Complement C2 | p<0.05 | 2.7 | 0.09 | 0.04 | Elevated in T2D vs Control | |
|  | Factor B | p<0.05 | 22 | 0.08 | 0.04 | Elevated in T2D vs Control | |
|  |  |  |  |  |  |  |  |
| **HYPOGLYCEMIA (HYPO)** | **T2D vs Control** | **p-value** | **% difference at HYPO** | **Fold difference at HYPO** | **Standard deviation (SD)** | **Direction** |  |
|  | Complement C2 | p<0.05 | 2.7 | 0.09 | 0.04 | Elevated in T2D vs Control | |
|  | Factor B | p<0.01 | 34.7 | 0.13 | 0.04 | Elevated in T2D vs Control | |
|  | Factor I | p<0.05 | 44.1 | 0.13 | 0.06 | Elevated in T2D vs Control | |
|  | Complement C4b | p<0.05 | 1.6 | 0.38 | 0.19 | Elevated in Control vsT2D | |
|  |  |  |  |  |  |  |  |
| **POST-HYPOGLYCEMIA** | **T2D vs Control** | **p-value** | **% difference POST-HYPO** | **Fold difference POST-HYPO** | **Standard deviation (SD)** | **Direction** |  |
| 4-hours | Complement C2 | p<0.05 | 2.9 | 0.10 | 0.04 | Elevated in T2D vs Control | |
| 30-minutes | Factor B | p<0.001 | 31.9 | 0.11 | 0.04 | Elevated in T2D vs Control | |
| 1-hour | Factor B | p<0.05 | 21.5 | 0.08 | 0.04 | Elevated in T2D vs Control | |
| 4-hours | Factor B | p<0.01 | 33.4 | 0.12 | 0.04 | Elevated in T2D vs Control | |
| 1-hour | Factor I | p<0.05 | 47.9 | 0.13 | 0.05 | Elevated in T2D vs Control | |
| 2-hours | Factor D | p<0.05 | 2.2 | 0.18 | 0.08 | Elevated in T2D vs Control | |
| 4-hours | Factor H | p<0.05 | 33.4 | 0.06 | 0.03 | Elevated in T2D vs Control | |
| 30-minutes | Mannose-binding protein C | p<0.05 | 48.3 | 0.28 | 0.16 | Elevated in T2D vs Control | |
| 2-hours | Properdin | p<0.05 | 199.6 | 0.13 | 0.06 | Elevated in Control vsT2D | |
| 30-minutes | Complement C3b | p<0.05 | 122.4 | 0.54 | 0.22 | Elevated in Control vs T2D | |
| 1-hour | Complement C3b | p<0.05 | 91 | 0.37 | 0.26 | Elevated in Control vs T2D | |
| 2-hours | Complement C3b | p<0.01 | 82.1 | 0.43 | 0.18 | Elevated in Control vs T2D | |
| 4-hours | Complement C3b | p<0.01 | 74.7 | 0.40 | 0.17 | Elevated in Control vs T2D | |
| 24-hours | Complement C3b | p<0.05 | 115.7 | 0.53 | 0.25 | Elevated in Control vs T2D | |
| 2-hours | Factor H-related protein 5 | p<0.05 | 1.4 | 0.14 | 0.07 | Elevated in Control vs T2D | |
| 1-hour | Complement C1q | p<0.05 | 48.3 | 0.20 | 0.08 | Elevated in Control vs T2D | |
| 2-hours | DAF | p<0.05 | 18.4 | 0.14 | 0.06 | Elevated in Control vs T2D | |
|  |  |  |  |  |  |  |  |
| **B. Within cohort differences over the study time course** | | |  |  |  |  |  |
| **POST-HYPOGLYCEMIA** | **CONTROL ONLY** | **p-value** | **% difference POST-HYPO** | **Fold difference POST-HYPO** | **Standard deviation (SD)** | **Direction** |  |
| 2-hours | Factor I | p<0.05 | 42.5 | 0.07 | 0.03 | Elevated versus hypoglycemia | |
| 2-hours | Factor D | p<0.05 | 2.8 | 0.20 | 0.08 | Decreased versus baseline | |
| 2-hours | Complement C4a | p<0.05 | 120 | 0.09 | 0.04 | Decreased versus baseline | |
| 2-hours | Complement C5 | p<0.05 | 28.3 | 0.22 | 0.05 | Decreased versus baseline | |
|  |  |  |  |  |  |  |  |
| **POST-HYPOGLYCEMIA** | **T2D ONLY** | **p-value** | **% difference POST-HYPO** | **Fold difference POST-HYPO** | **Standard deviation (SD)** | **Direction** |  |
| 2-hours | Properdin | p<0.01 | 300.4 | 0.18 | 0.06 | Decreased versus baseline | |
| 2-hours | DAF | p<0.01 | 21.8 | 0.15 | 0.04 | Decreased versus baseline | |
| 2-hours | Complement C3d | p<0.01 | 40.1 | 0.30 | 0.10 | Decreased versus baseline | |
|  |  |  |  |  |  |  |  |
| **POST-HYPOGLYCEMIA** | **T2D and CONTROL** | **p-value** | **% difference POST-HYPO** | **Fold difference POST-HYPO** | **Standard deviation (SD)** | **Direction** |  |
|  | **T2D** |  |  |  |  |  |  |
| 30-minutes | Complement C3b | p<0.01 | 87 | 0.41 | 0.07 | Decreased versus baseline | |
| 1-hour | Complement C3b | p<0.001 | 107.5 | 0.65 | 0.11 | Decreased versus baseline | |
| 2-hours | Complement C3b | p<0.0001 | 145.7 | 0.88 | 0.08 | Decreased versus baseline | |
| 4-hours | Complement C3b | p<0.0001 | 139.6 | 0.74 | 0.11 | Decreased versus baseline | |
| 1-hour | Complement C1r | p<0.05 | 5.0 | 0.19 | 0.07 | Decreased versus baseline | |
| 2-hours | Complement C1r | p<0.0001 | 14.2 | 0.71 | 0.09 | Decreased versus baseline | |
| 4-hours | Complement C1r | p<0.0001 | 7.7 | 0.32 | 0.07 | Decreased versus baseline | |
| 24-hours | Complement C1r | p<0.01 | 7.0 | 0.32 | 0.08 | Decreased versus baseline | |
| 30-minutes | Complement C5a | p<0.05 | 21.3 | 0.16 | 0.04 | Decreased versus baseline | |
| 1-hour | Complement C5a | p<0.05 | 23.4 | 0.21 | 0.03 | Decreased versus baseline | |
| 2-hours | Complement C5a | p=0.0001 | 38.7 | 0.37 | 0.05 | Decreased versus baseline | |
| 4-hours | Complement C5a | p<0.05 | 27.0 | 0.21 | 0.04 | Decreased versus baseline | |
| 2-hours | Complement C5b-C6 complex | p<0.001 | 1.8 | 0.24 | 0.04 | Decreased versus baseline | |
|  |  |  |  |  |  |  |  |
|  | **CONTROL** |  |  |  |  |  |  |
| 2-hour | Complement C3b | p<0.001 | 183.6 | 0.69 | 0.14 | Decreased versus baseline | |
| 4-hour | Complement C3b | p<0.001 | 184.9 | 0.64 | 0.14 | Decreased versus baseline | |
| 2-hours | Complement C1r | p<0.001 | 8.9 | 0.46 | 0.10 | Decreased versus baseline | |
| 4-hours | Complement C1r | p=0.001 | 8.1 | 0.38 | 0.09 | Decreased versus baseline | |
| 2-hours | Complement C5a | p=0.05 | 28.3 | 0.22 | 0.05 | Decreased versus baseline | |
| 2-hours | Complement C5b-C6 complex | p<0.01 | 1.2 | 0.15 | 0.04 | Decreased versus baseline | |
